# Diastolic dysfunction in patients with preserved ejection fraction: identification by velocity encoded magnetic resonance imaging

**DOI:** 10.1186/1532-429X-13-S1-P330

**Published:** 2011-02-02

**Authors:** Ulf K Radunski, Kai Muellerleile, Yasmin Meier, Christian R Habermann, Dietmar Koschyk, Ralf Koester, Gunnar K Lund, Gerhard Adam, Thomas Meinertz, Achim Barmeyer

**Affiliations:** 1 University Medical Center Hamburg-Eppendorf, Hamburg, Germany

## Purpose

To evaluate the ability of velocity encoded (VENC) magnetic resonance imaging (MRI) to identify patients with diastolic dysfunction in comparison with Doppler-echocardiography.

## Introduction

Diastolic dysfunction was recently recognized as an important cause for heart failure in patients with preserved ejection fraction. Diastolic dysfunction is typically assessed in clinical routine using transmitral and pulmonary-venous flow characteristics by Doppler-echocardiography. We hypothesized that VENC-MRI has a similar ability to identify patients with diastolic dysfunction compared to Doppler-echocardiography.

## Methods

The study included 76 patients with normal systolic left-ventricular function and a high probability of diastolic dysfunction. Transmitral flow and pulmonary venous flow profiles were obtained by VENC-MRI and Doppler-echocardiography. Measurements included maximal early- and late-diastolic transmitral velocities (E- and A-waves) as well as maximal systolic and diastolic pulmonary venous velocities (S- and D-wave). Furthermore, VENC-MRI was used to assess total diastolic left ventricular (LV) filling as well as the early and late portions of diastolic LV filling.

## Results

**Figure 1:**
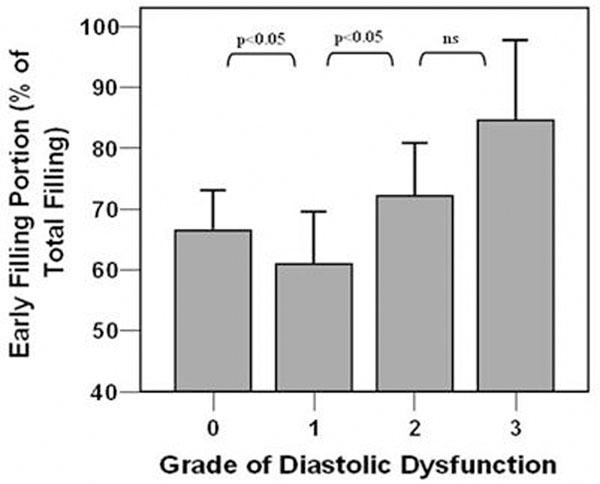


Doppler-echocardiography identified 38 patients with diastolic dysfunction. 24 patients had grade I diastolic dysfunction, 8 patients hat grade II diastolic dysfunction and 3 patients had grade 3 diastolic dysfunction. There was a very good correlation between VENC-MRI and Doppler-echocardiography for the E/A-ratio using peak velocities (r=0.73, P<0.0001). A moderate correlation was found for the S/D-ratio between VENC-MRI and Doppler-echocardiography using peak velocities (r=0.54, P<0.01). In grade I diastolic dysfunction, the contribution of early LV filling to total LV filling was significantly lower compared to patients with normal diastolic function or higher grades of diastolic dysfunction (Figure 1).

## Conclusions

VENC-MRI has the ability to identify diastolic dysfunction by measurements of transmitral flow velocities. Furthermore, the assessment of early and late LV diastolic filling volumes may provide additional information for the grading of diastolic dysfunction.

